# The impact of distance cataract surgical wet laboratory training on cataract surgical competency of ophthalmology residents

**DOI:** 10.1186/s12909-021-02659-y

**Published:** 2021-04-19

**Authors:** Amelia Geary, Qing Wen, Rosa Adrianzén, Nathan Congdon, R. Janani, Danny Haddad, Clare Szalay Timbo, Yousuf M. Khalifa

**Affiliations:** 1 Orbis International, 520 8th Avenue, Floor 12, New York, NY 10018 USA; 2 Centre for Public Health, Queen’s University Belfast, Belfast, BT12 6BJ Northern Ireland; 3 Instituto Regional de Oftalmología Javier Servat Univazo, Trujillo, La Libertad Peru; 4 Universidad Nacional de Trujillo, Trujillo, La Libertad Peru; 5 Aravind eye hospital, Madurai, Tamil Nadu 625020 India; 6 Emory Eye Center, Emory University, Atlanta, GA USA

**Keywords:** Cataract surgical training, Ophthalmic residency training, Simulation, Mentorship, Distance-learning, COVID – 19

## Abstract

**Background:**

This study assessed the impact of distance cataract surgical wet laboratory training on surgical competency of ophthalmology residents at a tertiary-level ophthalmic training center in Trujillo, Peru.

**Methods:**

Three five-week distance wet lab courses were administered through Cybersight, Orbis International’s telemedicine platform. Weekly lectures and demonstrations addressed specific steps in phacoemulsification surgery. Each lecture had two accompanying wet lab assignments, which residents completed and recorded in their institution’s wet lab and uploaded to Cybersight for grading. Competency was assessed through anonymous grading of pre- and post-training surgical simulation videos, masked as to which occurred before and after training, using a standardized competency rubric adapted from the Ophthalmology Surgical Competency Assessment Rubric (OSCAR, scale of 0–32). Day one best-corrected post-operative visual acuity (BVCA) was assessed in the operative eye on the initial consecutive 4–6 surgeries conducted by the residents as per the norms of their residency training. An anonymous post-training satisfaction survey was administered to trainees’.

**Results:**

In total, 21 ophthalmic residents participated in the courses, submitting a total of 210 surgical videos. Trainees’ average competency score increased 6.95 points (95%CI [4.28, 9.62], SD = 5.01, *p* < 0.0001, two sample t-test) from 19.3 (95%CI [17.2, 21.5], SD = 4.04) to 26.3 (95%CI [24.2, 28.3], SD = 3.93). Visual acuity for 92% of post-training resident surgeries (*n* = 100) was ≥20/60, meeting the World Health Organization’s criterion for good quality.

**Conclusions:**

Structured distance wet lab courses in phacoemulsification resulted in significantly improved cataract surgical skills. This model could be applicable to locations where there are obstacles to traditional in-person training, such as the current COVID-19 pandemic.

## Background

Lack of human resources in eye care delivery is a global problem. At present, there are not enough ophthalmologists capable of performing high-quality cataract surgery to meet the need, which is growing due to expansion and aging of the global population [1, 2]. The burden of unoperated cataract is unfairly borne by populations of low and middle-income countries (LMICs), where the lack of trained ophthalmologists is most acute [3]. Among existing ophthalmologists in low-resource settings, many do not operate at all, or have low surgical output [4]. Additionally, visual outcomes of cataract surgery in many LMICs fail to meet the World Health Organization’s (WHO) recommended standards [5].

This lack of ophthalmologists is not limited to low-income countries. While a third of ophthalmologists worldwide practice in the USA, Russia, and China and there is a global rise in the total number, the growing and aging population in many high-income countries is growing faster than the number of ophthalmologists. These aging populations will put pressure on current ophthalmic service providers globally, resulting in challenges in training enough doctors in the coming years [1].

While training is crucial to meeting the growing need for surgical services in eye care [6], the majority of residents in many settings, including those at high volume and well-staffed teaching hospitals, are not receiving adequate hands-on surgical training to ensure competency in cataract surgery [7], with some receiving none at all [8, 9]. One approach to improved surgical skills and the delivery of hands-on training has been surgical simulation training via a wet/dry lab. Surgical simulation can reduce the learning curve of difficult surgical techniques, accelerate the rate for trainees to achieve surgical competency, and improve patient safety [10]. However, there are challenges to the delivery of surgical simulation training reported by both low/middle-income and high-income countries alike [11, 12], including the absence of structured simulation training programs and the lack of trained instructors or personnel [13].

The current paper reports on a novel and scalable model for cataract surgical training: distance wet labs. Data are presented on masked, anonymous assessments of surgical videos before and after training, to determine whether this approach could lead to objectively measurable improvements in surgical competency.

## Methods

The La Libertad region of Peru is the third most populous in the country, with 1,617,050 inhabitants, 50% of whom live in the capital city of Trujillo. The majority of heath care workers in the Peru Northern zone (population approximately 4.5 million) are based in Trujillo [14]. The regional population is aging rapidly due to a reduction in the fertility rate and rising life expectancy [15], increasing the number at risk for cataract blindness. Some 58% of the estimated 150,000 blind persons in Peru have lost sight from unoperated cataract, and cataract surgical coverage is only 63% [16].

In 2017, Orbis International partnered with the Instituto Regional De Oftalmología Javier Servat Univazo (IRO) in Trujillo to pilot new methods of delivering cataract surgical training, which could be added to their existing resident training norms and residency curriculum. IRO is staffed by 25 ophthalmologists and 17 residents, serves as the main eye care referral center for La Libertad and the Northern Peru Zone and is the second largest training institute in ophthalmology in the country which accepts international residents from throughout Latin America. In 2016, the hospital had 135,969 outpatient visits, and performed 6841 surgeries, of which, 2254 were cataract surgeries [17]. IRO was also the first teaching hospital in Latin America to receive the International Council of Ophthalmology Accreditation as an “Advanced Surgical Training Program.”

Despite the fact that IRO is a busy hospital with a large faculty, residents were not receiving adequate cataract surgical training in phacoemulsification. Some of the reasons shared by the faculty were: high clinic volume keeping both faculty and residents occupied, the majority of senior faculty are unavailable in the afternoons, when they work in their private sector to supplement low government wages (~$1000/month) and therefore not readily available for training, and residents only start practicing phacoemulsification in their final year, limiting the number of cases they receive. These same issues are seen in other large hospitals, where consultants invest minimal time in training residents and busy clinics keep residents occupied, leaving little additional time for wet lab training [18].

As part of this partnership, Orbis and IRO began delivering structured distance wet lab courses in phacoemulsification for residents to improve their cataract surgical skills. No other changes to the residency curriculum were made besides the introduction of the five-week wet lab training in phacoemulsification. Orbis partnered with Emory University in Atlanta, USA, and IRO to deliver three distance wet lab surgical courses targeting residents in their second or final years of training. The first course was held in January–February 2017 for five residents, the second course in October–November 2017 for 7 residents and the third course in October 2018 for 9 residents. A structured course was developed to mimic the in-person wet lab training delivered to Emory ophthalmology residents. The training was administered by a mentor from Emory Eye Center (YK) and a course director at IRO (RA), and was delivered through Cybersight, Orbis’ telemedicine and learning platform, which is hosted on a mobile responsive learning management system (LMS). The LMS allows for curriculum creation, interactivity between learners and mentors, task assignment, and the grading and tracking of learners’ progress and results. Ethics approval was obtained from the Institutional Review Board at IRO, written consent was obtained from the leadership of IRO and the tenets of the Declaration of Helsinki were followed throughout.

The residency program at IRO introduces phacoemulsification surgery in the final year of training. Second year residents practice manual small incision cataract surgery or extracapsular cataract extraction. Therefore, final year residents were selected for the first and second distance wet lab courses. For the third course, second year residents were also included, as IRO faculty decided they wanted residents to participate in the course twice, once in the second and once in their final year of residency. All residents were new to phacoemulsification surgery when enrolled in the course, having no prior experience performing such cases in the operating room.

Prior to the course, an orientation was given to residents to familiarize them with Cybersight, including information on registering for and accessing the course; recording and uploading assignments; initiating online communication with the mentor; and obtaining other available open-source educational materials. Remote testing of the internet speed and bandwidth in Trujillo was completed by the information technology staff at IRO and Orbis. A minimum Internet connection of 3Mbps upload and download is desirable for real-time video and uploading recorded wet lab video assignments, similar to the requirements for watching YouTube videos. However, it is possible to use systems that can adapt to lower bandwidth/slower internet connections, which Orbis has deployed in other project locations.

The course was designed to run for 5 weeks, with each week addressing specific, sequential steps in phacoemulsification surgery (Table 1). The mentor delivered weekly lectures and demonstrations by live video conference. Conferences were interactive, with opportunities for questions, quizzing of students and repetition of demonstrations when requested. Each lecture had two accompanying wet lab assignments, for a total of 10 assignments. Residents had 4 days to practice, complete and record their assignments on video in the IRO wet lab. Practice was self-paced and determined by the resident. Assignments were conducted on either animal eyes, Kitaro® Wet Lab kit (FCI Ophthalmics, Marshfield Hills, MA., USA) simulation eyes or Philips Studio artificial eyes (Bristol, UK). With the first course, sessions 1 and 2 were performed with animal eyes and the remaining three sessions with model eyes. In the second and third course, all sessions were done with model eyes with the exception of session one.

**Table 1:** Tele-medicine Phacoemulsification Wet Lab Cataract Surgery Course

Week	Model	# of Eyes per Resident	Topic and Specific Steps in the OSCAR Rubric	Video Assignment
0	Artificial Eye	3 eyes each	Pre-course videos	Upload three complete Phaco simulation cases to Cybersight per participant
1	Pig Eye	5 eyes each	*Competency 1 & 2* Paracentesis, Keratome, Cannula	**Video One:** 20 Paracenteses, and enter with cannula through paracentesis port 20 times **Video Two:** 20 Keratome incisions
2	Artificial Eye	1 eye each plus sufficient film to practice	*Competency 3 & 4* Centration, Capsulorhexis	**Video Three:** 30 repetitions of dominant and non-dominant hand instrument movement in the anterior chamber **Video Four:** 10 Capsulorhexis
3	Artificial Eye	10 eyes each	*Competency 5 & 6, 14* Hydrodissection, Nucleus rotation, Grooving	**Video Five:**10 Hydrodissections; 10 Nucleus rotations in the bag; **Video Six:** 5 Maltese Crosses to 80% depth
4	Artificial Eye	7 eyes each	*Competency 7, 8, 9, 10, 15* Nucleus Disassembly, Quadrant Removal	**Video Seven:** 3 Nucleus disassembles with quadrant removal **Video Eight:** An additional 2 nucleus disassembles with quadrant removal
5	Artificial Eye	3 eyes each (can reuse from the previous week)	*Competency 11, 12, 13, 16* Irrigation/Aspiration, intra-ocular lens insertion	**Video 9:** 5 Use of irrigation/aspiration **Video 10:** 3 intra-ocular lens insertions in the bag
6	Artificial Eye	3 eyes each	Post-course videos	Upload three complete Phacoemulsification simulation cases to Cybersight per participant

Once completed, residents uploaded their video assignments to the Cybersight platform for grading by the course mentor (YK). The system automatically prompted the mentor to review and grade each resident’s assignment directly on the Cybersight platform. All grading was done against the Ophthalmology Simulated Surgical Competency Assessment Rubric (OSSCAR) [19], a standardized competency rubric adapted from the International Council of Ophthalmology’s Ophthalmology Surgical Competency Assessment Rubric (ICO-OSCAR) [20].

The OSSCAR (Table 2) has 16 steps, compared to the ICO-OSCAR, which has 20 steps. Steps not appropriate for or considered too advanced for the wet lab setting were removed, including step 1 on draping, step 15 on minimizing eye rolling and corneal distortion, step 18 on intraocular spatial awareness and step 19 on iris protection. Other steps were combined, such as step 7 and 8 on use of the phacoemulsification probe and second instrument; and some steps were broken down into two parts, such as step 11 on chopping and phacoemulsification of the nucleus. All 16 steps of the OSSCAR were covered in the course. The OSSCAR has three possible grades (0–2) for each surgical step, corresponding respectively to Novice, Advanced Beginner and Competent (total grading scale 0–32). The ICO-OSCAR by comparison has four grades (2–5), corresponding to Novice, Beginner, Advanced Beginner and Competent (total grading scale 0–100).

**Table 2:** Ophthalmic Simulated Surgical Competency Assessment Rubric (OSSCAR)– Phacoemulsification

		Novice (score = 0)	Advanced Beginner (score = 1)	Competent (score = 2)	Score (Not done score = 0)
1	**Incision and paracentesis formation technique**	Poor wound construction and paracentesis placement. Traumatizes conjunctiva	Correct positioning of incision and paracentesis but incision architecture is not yet correct	Well-constructed incision and paracentesis with careful tissue handling	
2	**Viscoelastic: appropriate use and safe insertion**	Incomplete fill +/− damage to capsule	Appropriate fill but still hesitant	Safe and smooth insertion of viscoelastic	
3	**Capsulorhexis: Commencement of flap**	Poor positioning of initial flap with disruption of underlying cortex	Good positioning of flap but slightly hesitant in raising the flap	Neat creation of a flap of an appropriate size in the correct position.	
4	**Capsulorhexis: Formation and circular completion**	Unable to create a complete capsulorhexis with poor understanding of tearing vectors	Capsulorhexis is completed but is either too small, too large or eccentric	Smooth creation of an appropriately sized and circular capsulorhexis	
5	**Hydrodissection: visible fluid wave and free nuclear rotation**	Cannot insert cannula in the correct tissue plane / excessive or insufficient force used / incomplete freeing of the nucleus	Cannula inserted correctly under the anterior capsule but more than one attempt is needed to achieve free nucleus rotation	Efficient and safe hydrodissection with free nuclear rotation	
6	**Phaco probe and second instrument: effective use and stability within the eye**	Unsure of the positioning of the instruments within the eye / phaco probe is frequently close to the capsulorhexis / inefficient use of the second instrument	Phaco probe and second instrument generally positioned correctly / no iris trauma / capsulorhexis not endangered	Confident instrument handling with phaco probe always kept in a safe position	
7	**Nucleus: sculpting or primary chop**	Hesitant use of the phaco probe / tendency to push the lens / timid sculpting with poor use of full range of phaco power	More efficient use of phaco power and appropriate vacuum settings to create a groove or perform a primary chop / still some stress placed on zonules	Fast and efficient sculpting or chopping technique	
8	**Nucleus: Rotation and manipulation**	Incorrect positioning of the instruments / excessive posterior pressure on the lens / rounds off the edges of the quadrants leaving a bowl	Good positioning of instruments but still some hesitancy using the second instrument / some posterior pressure whilst rotating the nucleus	Confident use of both phaco probe and second instrument to rotate the lens with no posterior pressure on the zonules	
9	**Nucleus: cracking or chopping**	Attempts to crack the lens before groove is deep enough / places instruments too superficially in the groove / excessive posterior pressure during cracking	Forms a grove of the correct depth and width before cracking / still requires several attempts to crack the nucleus	Good groove construction and cracks / chops nucleus at first attempt	
10	**Nucleus: segment removal**	Chases segments with phaco probe / poor use of the second instrument / endangers capsule / phaco probe positioned too close to posterior capsule or endothelium	Appropriate use of vacuum to engage segments / second instrument being used more efficiently / less of a tendency to phaco too deep in capsular bag or too close to the endothelium	Safe engagement of nuclear segments and efficient removal with good use of the second instrument	
11	**Irrigation and aspiration technique with adequate removal of cortex**	Aspiration port not safely positioned in the capsular bag / inappropriate vacuum used / hesitant engagement of cortex	Better positioning of aspiration port / still not using vacuum efficiently / occasionally engages the anterior capsule	Efficient removal of the cortex with no danger to the capsular bag or capsulorhexis	
12	**Lens insertion, rotation and final position of IOL**	IOL not placed in the capsular bag / unable to rotate the lens into the correct position	IOL placed in the capsular bag but haptics still require manipulation	IOL completely placed within the capsular bag at the first attempt	
13	**Wound closure: hydration, suturing if required and checking security**	Ineffective hydration technique / does not check would security / poor placement and tying of 10/0 suture	Wound hydration performed correctly / suture tying hesitant / suture slightly too tight or too loose	Wound hydration performed correctly / good suturing technique with correct tension	
**Global Indices**					
14	**Tissue handling:**	Tissue handling is often unsafe with inadvertent damage to the conjunctiva, cornea, iris or capsule/excessively aggressive or timid.	Tissue handling is safe but sometimes requires multiple attempts to achieve desired manipulation of tissue.	Tissue handling is efficient, fluid and almost always achieves desired tissue manipulation on first attempt.	
15	**Eye positioning and use of the microscope**	Eye is frequently in an eccentric position. Focusing and X-Y movement of the microscope is erratic.	Eye is mainly kept in a central position and focusing of the microscope is becoming smoother.	Eye is maintained in a central position throughout the procedure and the point of interest is always in focus.	
16	**Overall speed and fluidity of the procedure**	Hesitant and lacks fluidity with multiple pauses between maneuvers	Beginning to string the different steps together with minimal guidance from trainer	All steps completed in a timely manner with minimal input from trainer	

A passing score of 1 on the 0–2-point scale (“Advanced Beginner”) for each surgical step was necessary to continue to the next step; anything less required the resident to redo and resubmit the assignment. Residents could access their grades and communicate directly with the course mentor via the Cybersight platform, receiving unstructured, informal feedback in an ongoing manner for each assignment and on their overall performance.

Upon completion of the course, the course director and residency director received a comprehensive digital report generated by Cybersight on each resident’s weekly performance, including their grades for each assignment. Residents who successfully completed the course received a certificate of completion, automatically generated by Cybersight.

Orbis tracked the surgical outcomes of first consecutive 4–6 phacoemulsification surgeries conducted by participating residents after they completed the course. Orbis also retrospectively collected the surgical outcomes for the first 4–6 surgeries performed by senior year residents in the 2 years prior (2015–2016) to the first distance wet lab surgical course, to measure the training impact on residents’ real-world performance. Hands-on surgical training was conducted as per the norms of the residency program, with all surgeries being performed under the supervision of experienced consultants. Management of complications was done per partners’ clinical norms and standards. Nurse technicians measured and recorded the best-corrected day one post-operative visual acuity in the operative eye. We did not exclude results of patients with systemic or ocular comorbidities but took the first cases in sequential order for each resident performing cataract surgery using phacoemulsification after training. This reflects practice patterns at IRO where residents perform many surgeries on cases of patients with comorbidities.

Also, a self -administered course satisfaction survey developed by the investigators was shared with all resident participants to capture their feedback on course content, format, acceptability and self-assessment of improvements in their skill. Learners rated their level of agreement on a 5-point Likert scale, with 1 representing strongly disagree and 5 representing strongly agree. The survey was administered anonymously, using the Typeform platform (Typeform, Barcelona, Spain).

In addition, adjustments were made following the piloting of the first course, and the second and third courses (which enrolled 16 residents) included two further assignments, asking residents to upload three pre and three post-training cataract surgical simulation videos made using model eyes and covering all steps of the OSSCAR. Pre-course videos were uploaded within the week prior to beginning the course and post-course videos within 1 week after course completion. These videos were sent for anonymous grading using the OSSCAR competency rubric. The grader (RJ), an experienced ophthalmic surgeon, was masked as to which videos were recorded before and after training.

### Statistical methods

The difference between pre and post-course video overall grading was analyzed using the two-tailed paired sample t-test. To evaluate the impact of the training course on specific surgical steps assessed using OSSCAR, the Wilcoxon signed-rank tests were performed for each of the 16 MSIC surgical steps pre and post course. The training impact on the quality of surgical outcomes pre and post course introduction, was analysed using a two-proportion test. All statistical analyses were done in R version 3.6.1 (2019-07-05) (The R Foundation; Vienna, Austria).

## Results

We did not find significant differences in the performance of second- and third-year residents participating in the remote wet labs and as such, have combined their results. In total, 21 residents participating in the two courses, submitted a total of 210 surgical videos. A score of ≥1 was achieved on 205 (97.6%) assignments on the first submission, and 21 certificates of completion (100%) were awarded. A post-course satisfaction survey reported that 100% of residents would recommend the training to others, and all confirmed a desire to participate in similar future distance surgical mentorship wet labs.

Visual acuity was measured for 100 post-training resident phacoemulsification surgeries (one second-year resident has not yet performed phacoemulsification cases due to being on an outreach rotation), of which 92 (92%) had day one post-operative best corrected VA ≥20/60, meeting the WHO criterion for cataract surgical quality [21]. Once again, we did not find significant variations between the three wet lab classes, in their post-operative outcomes (range 91–93% best corrected visual acuity (BCVA) ≥20/60). We analyzed patient outcomes of the first phacoemulsification surgeries (*N* = 44) performed by residents (*N* = 9) in 2015 and 2016, prior to when the distance surgical wet lab training began and found that 75% (33) had a day one post-operative BCVA ≥20/60. This represents a 22.7% relative improvement in surgical outcomes, (an absolute increase of 17% (95% CI 1.5–32.5%, *P* = 0.012, two-proportions test)) for second and final year residents having taken the course over final year residents in the previous 2 years.

**Fig. 1:**
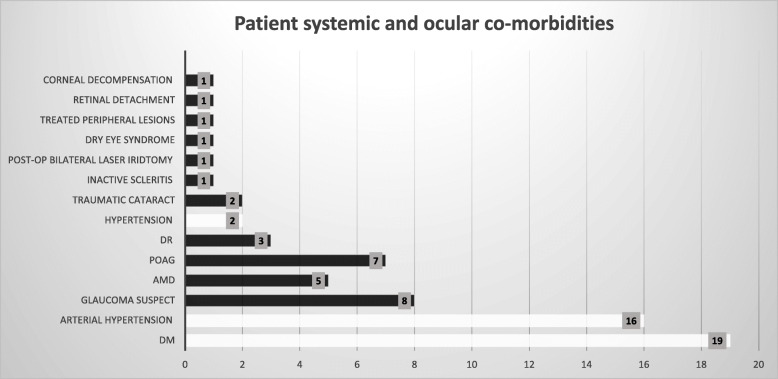
Patient systemic and ocular co-morbidities

**Fig. 2:**
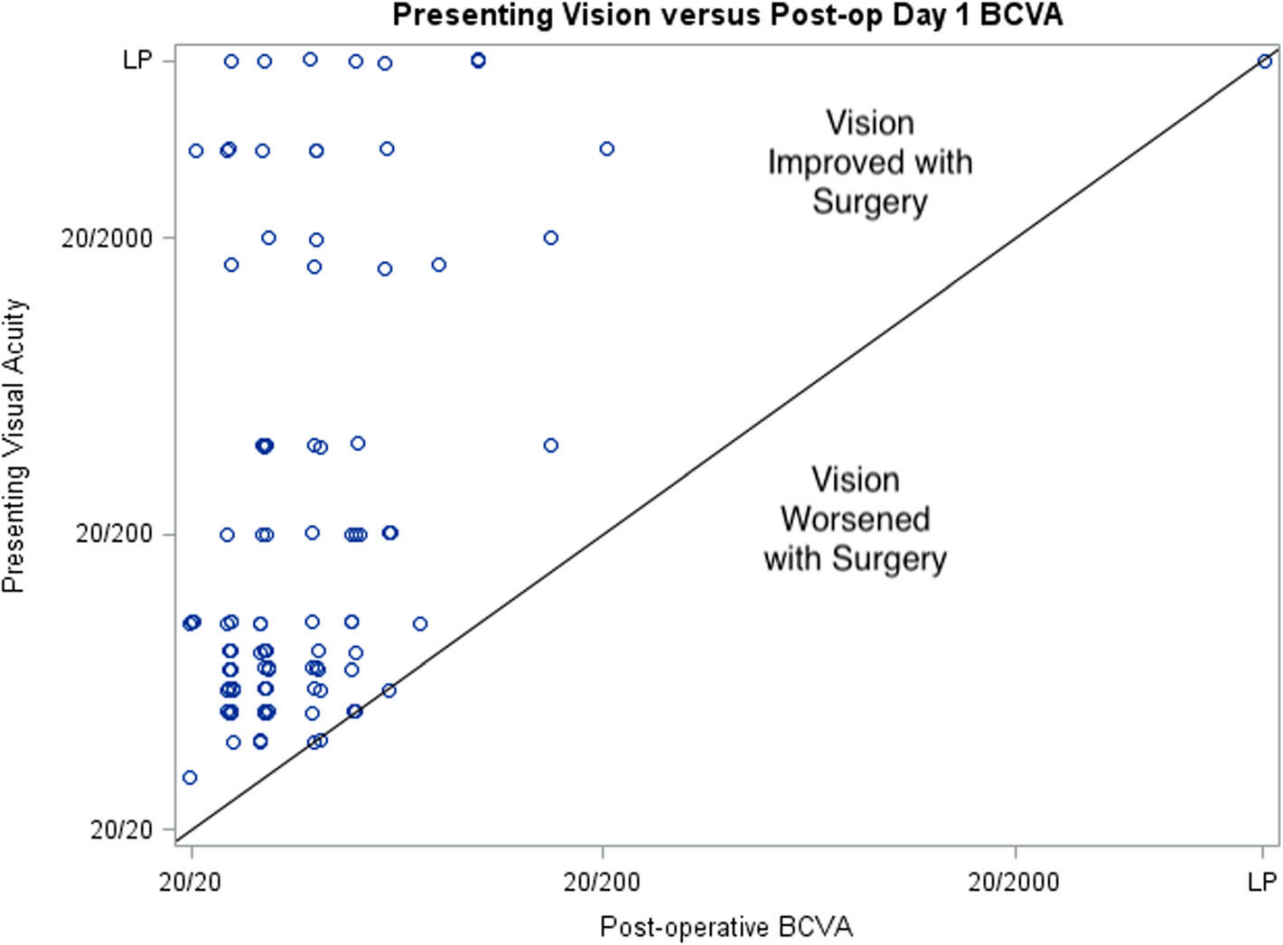
Presenting visual acuity versus post-operative day one best corrected visual acuity (BCVA)

Patients ranged in age from 19 to 95 years, with the mean (standard deviation) age being 70 (11.9) years, and 42 (42.0%) of the patients were men. A total of 55 (55.0%) patients presented with one or more systemic or ocular comorbidity, most commonly diabetes (*n* = 19, 19.0%), hypertension (*n* = 16, 16.0%), and suspected glaucoma (*n* = 8, 8.0%) (Fig. 1). BCVA ranged from light perception to 20/30, with a median of 20/100 and inter-quartile range of (20/60, 20/400), while comparable values for post-operative visual acuity in the operative eye were light perception to 20/20; median: 20/30; inter-quartile range (20/25, 20/50). (Fig. 2). Unfortunately, existing medical records did not document the type of cataract in a fashion that we felt was sufficiently consistent for analysis and publication.

Sixteen residents each uploaded three pre and three post-training videos for anonymous grading following the second and third course, for a total of 96 videos. Among these, 95 (99.0%) were graded, due to the low image quality of one pre-course video. The mean score was 26.3 (95%CI [24.2, 28.3], SD = 3.93) for the post-course videos grading, an increase of 6.95 (95%CI [4.28, 9.62], SD = 5.01, *p* < 0.0001, two sample t-test) compared to the mean score of 19.3 (95%CI [17.2, 21.5], SD = 4.04) for pre-course videos.

Eight out of 16 total surgical steps (50%) showed significant improvement (*p* < 0.05, two-sided Wilcoxon signed-rank test). The steps showing greatest improvement (*P* < 0.007, effect size r > 0.70) were: Lens insertion, rotation and final position of intraocular lens (IOL), *P* = 0.000381, median (pre-course: 0, post-course: 1.67), effect size = 0.895; irrigation and aspiration technique with adequate removal of cortex *P* = 0.000438, median (pre-course: 0, post-course: 2), effect size = 0.886; and capsulorhexis formation and circular completion *P* = 0.00628, median (pre-course:1.58, post-course: 2), effect size = 0.721.

## Discussion

Participation in the course was associated with a significant improvement in participating residents’ cataract surgical scores. This is consistent with Dean et al.’s multicentre, parallel-group randomised clinical education-intervention trial, which measured cataract surgical competency using a similar modified ICO-OSCAR. This study demonstrated statistically significant improved surgical competency after a 5-day intensive simulation training course using model eyes for the intervention group and between the intervention and control group. The difference in OSSCAR scores was 16.6 points higher in the intervention group (95% CI, 14.4–18.7) with adjustment for baseline scores (*P* < .001) [22].

Post course surgical visual outcomes met WHO standards for cataract surgical quality. IRO and Orbis have now agreed to make this course a permanent part of residency training, with all second- and final-year residents participating annually in October. Distance wet lab mentorship courses are currently being replicated at Orbis partner hospitals in Bolivia and Haiti, teaching the manual small incision cataract surgical technique. Based on the positive user feedback, it appears that structured distance wet lab courses in phacoemulsification are acceptable to ophthalmologists in training.

Tele-education or distance learning is widely used in academic training today. By 2006–07, 61% of US higher education institutions offered some form of online courses [10]. It is likely that this number has continued to rise, as online education provides greater cost-efficiency and allows access to learners in remote locations [10]. There is evidence of better performance among students who engage in flipped classrooms or blended-learning, defined as approaches combining in-person and online activities. A group of medical students at Zhongshan Ophthalmic Center who received online lectures and resources during their ophthalmology clerkships performed better on subsequent examinations and reported improved motivation and understanding of the material in comparison to a group who attended traditional classroom-based lectures [11]. The University of Miami Miller School of Medicine integrated flipped courses for students of ophthalmology, after which students performed better in examinations than in four previous semesters where typical teaching methods were applied [23]. Additionally, ophthalmologists recruited in Brazil, Mexico and the Philippines who were trained in the diagnosis of retinopathy of prematurity through a structured tele-education course showed significantly-improved diagnostic accuracy and reliability, and reported a preference for tele-education to traditional teaching methods [12].

Wet lab education is also a common teaching method and found in many current ophthalmic residency programs. An ophthalmologist experiences the highest complication rates during the first 60–80 surgical cases [13, 14, 22], while evidence demonstrates that structured cataract surgical simulation courses can reduce the complication rates in these initial operations [16]. Simulation training of micro-surgical skills has shown improved performance outcomes [24]. Training using model eyes has shown improvements in both the accuracy and speed for surgically naïve residents in performing capsulorhexis and corneal sutures [25] and improvements in corneal suturing were also found with experienced cornea surgeons [26]. Dean et al.’s demonstrated that intense simulation based cataract surgical education using models eyes resulted in improved surgical competence, as well as improved live surgical performance and reduced surgical complication rates [22].

The use of the virtual reality cataract surgical simulator Eyesi (VRmagic, Mannheim, Germany) has also shown improved performance among cataract surgery trainees [27]. Eyesi cataract simulation training has shown improvements in resident ophthalmologists’ confidence in performing phacoemulsification [28]. A Royal College of Ophthalmologists’ National Ophthalmology database study of nearly 18,000 cases performed by first- and second-year residents showed a 38% reduction in posterior capsule rupture rates for residents who’d undergone simulation training compared to trainees who did not receive this form of simulation training [29]. However, the Eyesi system is cost prohibitive for many residency programs in LMICs, both in terms of initial purchase and annual costs to maintain the license and support contract, and therefore has not been widely adopted.

Combining these two effective teaching models by delivering wet lab training through distance learning, the program described in the current paper was able to realize improved performance outcomes.

Despite the effectiveness of simulation training, ophthalmic residents in low and middle-income countries, as well as high-income countries have inadequate simulation surgical courses [30, 31]. Some of the reasons reported were lack of structured or poorly organized simulation training programs; lack of simulation facilities; lack of trained instructors or personnel, disinterest in training residents from consultants, particularly for residents assigned to peripheral hospitals rather than teaching hospitals for clinical practice; high volume of residents due to government mandates, and outpacing the faculty available for teaching [32].

The model presented in this paper suggests that this approach is an effective way to improve the cataract surgical skills of trainees. Further, this model could provide a viable alternative method for delivering wet lab cataract surgical training in locations where some of the reported challenges to wet lab training, such as structured programs, limited faculty, and lack of access to training institutions, undermine the delivery of in-person structured wet lab courses.

Another consideration is the recent Corona virus disease (COVID-19) pandemic, which resulted in many ophthalmology departments closing or severely reducing their patient volume. This coupled with the need to practice social distancing, left many residents with limited access to traditional in-person hands-on training and clinical learning opportunities, essentially disrupting surgical training at a global level [33]. During COVID-19, many medical training institutions were forced to rethink how they delivered education, and explore the use of virtual teaching, distance learning and simulation, to meet the training needs of their learners [34]. Distance wet lab courses also offer the opportunity to provide continuity of training, respecting the need for social distancing, during disruptive events such as COVID-19. The University of California, San Francisco recently delivered a distance wet lab in corneal suturing, in which faculty and residents reported the session equally and/or more effective than previous in-person wet lab training in corneal suturing [35].

Regarding the difficulty of the different steps in phacoemulsification surgery, Dooley et al. [36] reported on 8 trainee surgeons’ assessment of the difficulty of 8 individual stages of phacoemulsification cataract surgery, after performing 100 consecutive surgeries. The trainees reported phacoemulsification and Continuous Curvilinear Capsulorhexis (CCC), as the most complicated stages, which also had the lowest completion rates (66.7 and 74.4% respectively). This was followed by irrigation/aspiration and IOL insertion, with completion rates of 78.4, and 83.1%, respectively. Similar results have been reported from mentors (evaluated) and trainees (self-assessed/reported), with phacoemulsification and CCC consistently reported as the most challenging steps to learn [37–39]. Our analysis showed that residents most significantly improved in lens insertion, rotation and final position of IOL, irrigation and aspiration technique with adequate removal of cortex and capsulorhexis-formation and circular completion. Five additional steps showed significant improvements (*p* < 0.05) including nucleus cracking or chopping. This indicates that the distance wet lab training is associated with improvements in intermediate to challenging surgical steps.

## Conclusions

Positive user feedback and the significant improvement in surgical performance based on masked assessment of videos by an experienced grader (RJ) suggest that distance wet lab mentorship courses can be an effective training tool in this setting. Further work is needed in other settings to confirm these results.

## Data Availability

The datasets used and/or analyzed during the current study are available from the corresponding author on reasonable request.

## Abbreviations

BCVA: Best Corrected Visual Acuity
CCC: Continuous Curvilinear Capsulorhexis
COVID – 19: Corona Virus Disease 2019
ICO: International Council of Ophthalmology
IOL: Intraocular Lens
IRO: Instituto Regional De Oftalmología
LMICs: Low- and Middle-Income Countries
LMS: Learning Management System
OSCAR: Ophthalmology Surgical Competency Assessment Rubric
OSSCAR: Ophthalmology Simulation Surgical Competency Assessment Rubric
WHO: World Health Organization
